# Congenital central diabetes insipidus and optic atrophy in a Wolfram newborn: is there a role for *WFS1* gene in neurodevelopment?

**DOI:** 10.1186/s13052-014-0076-4

**Published:** 2014-09-26

**Authors:** Stefano Ghirardello, Elisa Dusi, Bianca Castiglione, Monica Fumagalli, Fabio Mosca

**Affiliations:** Neonatal Intensive Care Unit Department of Clinical Sciences and Community Health, University of Milan, Fondazione IRCCS Ca’ Granda Ospedale Maggiore Policlinico, Milan, Italy

## Abstract

**Background:**

Wolfram syndrome (WS) is an autosomal recessive neurodegenerative disorder characterized by diabetes mellitus (DM), optic atrophy (OA), central diabetes insipidus (CDI) and deafness (D).

The phenotype of the disease has been associated with several mutations in the *WFS1 gene*, a nuclear gene localized on chromosome 4. Since the discovery of the association between *WFS1* gene and Wolfram syndrome, more than 150 mutations have been identified in WS patients.

We previously described the first case of perinatal onset of Wolfram syndrome newborn carrying a segmental uniparental heterodysomy affecting the short arm of chromosome 4 responsible for a significant reduction in wolframin expression.

Here we review and discuss the pathophysiological mechanisms that we believe responsible for the perinatal onset of Wolfram syndrome as these data strongly suggest a role for *WFS1* gene in foetal and neonatal neurodevelopment.

**Case presentation:**

We described a male patient of 30 weeks’ gestation with intrauterine growth restriction and poly-hydramnios.

During the first days of life, the patient showed a 19% weight loss associated with polyuria and hypernatremia. The presence of persistent hypernatremia (serum sodium 150 mEq/L), high plasma osmolarity (322 mOsm/L) and low urine osmolarity (190 mOsm/l) with a Uosm/Posm ratio < 1 were consistent with CDI. The diagnosis of CDI was confirmed by the desmopressin test and the brain magnetic resonance imaging (MRI) at 34 weeks of age, that showed the lack of posterior pituitary hyperintense signal. In addition, a bilateral asymmetrical optic nerve hypoplasia associated with right orbital bone hypoplasia was observed, suggesting the diagnosis of WF.

During the five years follow-up the patient did not developed glucose intolerance or diabetes mellitus. By the end of the second year of life, primary non-autoimmune central hypothyroidism and mild neurodevelopment retardation were diagnosed.

**Conclusions:**

The analysis of our case, in the light of the most recent literature, suggests a possible role for WFS1 gene in the development of certain brain structures during the fetal period.

Wolfram syndrome should be considered in the differential diagnosis of the rare cases of congenital central diabetes insipidus developed in the neonatal period.

## Background

Wolfram syndrome (WS1) (MIM #222300), first described by Wolfram and Wagener in 1938 [1], is a rare autosomal recessive neurodegenerative disorder with an estimated prevalence of 1: 770.000 live births and a carrier frequency of 1: 354 [2,3]. It is also known by the acronym DIDMOAD in order to include four characteristic clinical abnormalities: central diabetes insipidus (DI), diabetes mellitus (DM), optic atrophy (OA) and deafness (D).

The diagnosis of Wolfram syndrome is based on the presence of the two minimal diagnostic criteria: early onset DM (<15 years) and bilateral progressive OA. DM is usually the first symptom to present at a median age of 6 years, followed by the onset of OA at a median age of 11 years. The incidence of central DI varies considerably, ranging from 48% to 78%, as generally it does not appear until the 2^nd^ or 3^rd^ decade and, initially, it may be partial. Frequently, sensorineural deafness develops at an average age of 16 years (range 4–39 years). A wide spectrum of abnormalities of the endocrine glands, the central nervous system (CNS) and the urogenital tract has been described, comprising hypothyroidism, hypogonadism and ACTH deficiency, anosmia, ataxia, seizures, nystagmus, gaze palsies, dysarthria, dysphagia, psychiatric disorders, cognitive deficits, hypo or areflexia and neurogenic bladder, central sleep apnea, neurogenic upper airway collapse, myoclonus, Parinaud’s syndrome [3-7]. The leading cause of death, that typically occurs around the age of 30 (range 25–50), is the central respiratory failure resulting from brainsteam atrophy [7].

The disease phenotype has been associated to mutations in *WFS1*, a nuclear gene mapped on chromosome 4p16.1, encoding for the endoglycosidase membrane glycoprotein named wolframin. Wolframin plays a protective function against endoplasmic reticulum (ER) stress and it is involved in calcium storage, redox regulation, steroid synthesis, and cell death [8-12]. Acquired or inherited ER dysfunction can cause rare genetic diseases as well as common chronic diseases, including DM and neurodegenerative diseases [13-16].

Since the discovery of the association between *WFS1* gene and Wolfram syndrome, more than 150 mutations have been identified in WS patients. However, no evident genotype-phenotype correlations were found [7].

We recently described the first case of Wolfram syndrome perinatal onset, presenting with congenital OA and CDI, in a growth-restricted male infant, born at 30 weeks gestation,. A detailed description of the underlying genetic defect, a segmental uniparental heterodysomy affecting the short arm of chromosome 4 responsible for the significant reduction in wolframin expression, have been previously published [16].

Here we review the pathophysiological mechanisms possibly responsible for the perinatal onset of Wolfram syndrome according to the recent published evidence of *WFS1* role in fetal and neonatal neurodevelopment.

## Case report

We described a male patient, born to a 38 years old mother at 30 weeks’ gestation by emergency Cesarean section, performed because of pre-eclampsia and IUGR associated with poly-hydramnios, detected by 26 weeks. The pregnancy was complicated by maternal type 2 DM, requiring subcutaneous insulin, Antenatal serologies (TORCH, HIV, Treponema and Hepatitis) were protective. Cytogenetic investigation of the fetus by villocentesis confirmed a normal 46,XY karyotype. Paternal medical history was unremarkable. Parents were non-consanguineous Caucasians.

Apgar scores were 6 at 1 minute and 7 at 5 minutes of life. Physical parameters were as follows: weight 1190 g (<10° centile), length 36 cm (<3° centile) and head circumference 26 cm (<10° centile). Physical examination was normal except for a moderate respiratory distress, During the first hours of life, the respiratory status worsened, evolving towards a third-degree hyaline-membrane disease that required surfactant administration, mechanical ventilation for a 24 hour period and non-invasive ventilation for 10 days.

Throughout the first 3 days of life, the patient experienced a significant weight loss (as high as 19% of the birth weight), associated with polyuria (4.7-5.5 ml/kg/h) and hypernatremia (serum sodium 152–159 mEq/l), despite adequate fluid intake. At first, hypernatremia was interpreted as a consequence of the excessive weight loss and treated with large amounts of daily intravenous fluids (180–200 ml/kg/d). The clinical course was complicated by one episode of Serratia Marcescens septic shock during the second week of life and disseminated intravascular coagulation requiring plasma administration, platelet transfusion and aggressive antibiotic therapy.

CDI was subsequently suspected as a result of persistent hypernatremia (serum sodium 150 mEq/L), high plasma osmolarity (322 mOsm/L) and low urine osmolarity (190 mOsm/l) with a Uosm/Posm ratio < 1 associated with failure to thrive during the first four weeks of life. The diagnosis of CDI was confirmed by the desmopressin test, showing a normal urine concentrating capability after nasal desmopressin (DDAVP) administration with rapid raise in urine osmolarity and reduced plasma osmolarity and the brain MRI at 34 weeks postmestrual age demonstrating the lack of posterior pituitary hyperintense signal. Bilateral asymmetrical optic nerve hypoplasia associated with right orbital bone hypoplasia was also observed, suggesting the diagnosis of WF [17]. No other abnormalities of the CNS were found.

Ophthalmologic examination revealed eyeball and right optic disc hypoplasia, associated with exotropia. Visual evoked potential confirmed the pathological findings of the right optic pathway, while the electroretinogram resulted normal. Audiological examinations were normal. Borderline TSH values with normal thyroid function were recorded during hospitalization, but no treatment was required until the end of the first year of life, when levo-thyroxine substitutive therapy was started because of primary non-autoimmune central hypothyroidism with a small thyroid gland. During the five years follow-up mild psychomotor and mental retardation were diagnosed. No glucose intolerance or diabetes mellitus have been detected so far.

After the catch-up growth, length and head circumference has stabilized around the 50° centile and, after the first year of life, body weight was above the 97° centile.

Water and electrolyte balance is maintained with standard doses of nasal desmopressin.

No progression of the bilateral asymmetrical OA was noted at the 1-year follow-up MRI. However, new pathological signs (reduced corpus callosus, moderate ventricular dilatation, asymmetric hippocampus and altered occipital peri-ventricular white matter signal intensity) [17] were noticed.

Genetic investigation of the patient and his relatives revealed a segmental paternal heterodisomy of the chromosome 4 (segmental pat-hUPD4), involving at least part of the promoter and the first exon of *WFS1* that we hypothesized could remove splicing regulatory motifs located in intron 1. Direct sequencing analysis of *WFS1* cDNA showed a mixed sequencing pattern including the wild-type transcript sequence and two splice variants. The first splice variant was a previously described 4-bp deletion in exon 2, while the second one exhibited the complete skipping of exon 2 and, probably, the loss of the transcription start site. RT-PCR confirmed a normal mRNA expression compared to young controls, while Western blot analysis showed a significantly decreased Wolframin expression [17]. Informed consent for genetic studies was obtained from all subjects involved in the study.

## Discussion

In this report we deepened from the clinical point of view the case of an atypical Wolfram patient, presenting during fetal and neonatal life. As a matter of fact, we discovered DI and OA during the examination performed after the delivery, that occurred long before the term of pregnancy, albeit the antenatal origin of the CDI was suggested by poly-hydramnios, polyuria and rapid body-weight loss occurred since first days of postnatal life. In addition, we speculate that the OA and the hypoplasia of the eyeball were caused by a pathological process originating during fetal life.

**Figure 1:**
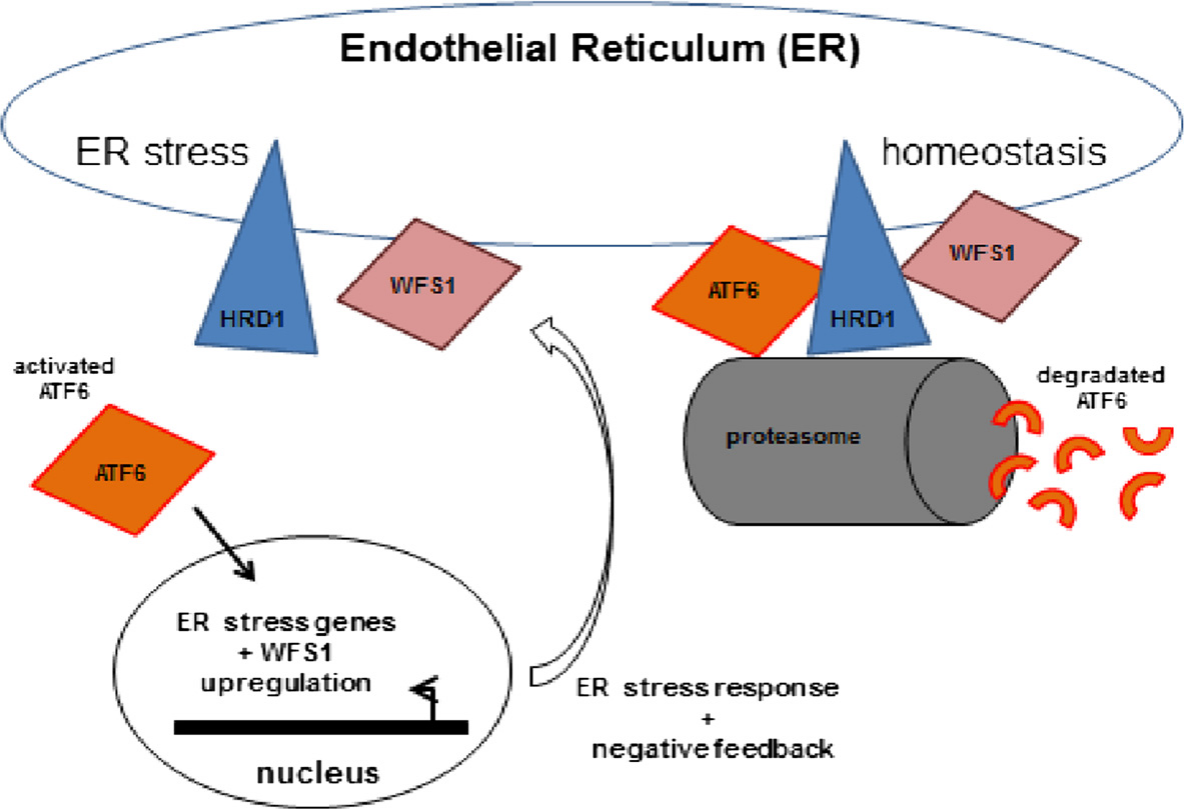
**WFS1 is a key element of the ER stress activated signalling cascade, called the unfolded protein response (UPR).** WFS1 regulates ATF6 protein through the ubiquitin-proteasone pathway, stabilizing and enhancing the function of its E3 ligase, HRD1 and acts as a negative regulator of this pathway. The E3 ubiquitin ligase HRD1 is found in the endoplasmic reticulum membrane and is involved in endoplasmic reticulum-associated degradation. In response to ER stress, the cleaved form of ATF6 translocates to the nucleus. The non-cleaved form of ATF6 is degraded by the ubiquitin-proteasome pathway to prevent the hyperactivation of the UPR.

The Wolfram syndrome is caused by mutations of the protein Wolframin, essential for the regulation of intracellular calcium homeostasis, the cell cycle progression and the retrieval of homeostasis under conditions such as ER stress [10-12]. Indeed, *WFS1* is a key element of the ER stress activated signalling cascade, also known as unfolded protein response (UPR). In particular, WFS1 regulates ATF6A activity, one of the ER stress-sensor molecules that induces UPR target genes and prevents cells from apoptosis caused by the imbalance between productive folding of secretory proteins and degradation of misfolded proteins [11,13] (Figure 1).

The ER environment can be disrupted by both, physiological (i.e. post-prandial insulin biosynthesis that increases the workload of the ER) and pathological (i.e. hypoxia, hypoglycaemia or mutated proteins) processes, causing the production of toxins, cytokines and mutant proteins expression [13,18,19].

Patients affected by Wolfram syndrome exhibit a dysregulated ER stress signaling. The uncontrolled ATF6α up-regulation determines apoptosis of sensitive cell types, in particular pancreatic β cells and neural cells [12,13].

Recent insight arising from animal studies led to the conclusion that Wolframin may also be essential for the processing of the arginine vasopressin (AVP)-precursor and for the survival of the supraoptic (SO) and the supraventricular hypothalamic (PV) nuclei neurons [20]. Gabreel and colleagues demonstrated the abnormal processing of AVP-precursor in the SO and PV nuclei and the presence of reactive gliosis in human tissues recovered during the autopsy of patients with longstanding CDI. On the contrary, no gliosis was detected in patients that were symptomatic for a shorter period of time, suggesting that the pathogenesis of CDI in Wolfram patients is characterized by a progressive three-step phase process: 1) a down-regulation of the amount of processed AVP and partial CDI, 2) a complete disappearing of processed AVP, and 3) a complete disappearance of AVP-producing cells in the hypothalamic nucleus [21].

Investigations in our patient did not allow neither to determine the mechanisms underlying the development of early onset CDI, nor to correlate his rare and previously undescribed mutation to his unique phenotype [17]. The most likely explanation is a specific and severe prenatal/postnatal ER workload in the SO and PVN, that led to a rapid neuronal dysfunction and/or death due to a very low Wolframin level. We speculated that the underlying mechanisms were the rising production of AVP during the second trimester of pregnancy (increase of over 1000 times), the active role of fetal vasopressin in the birth process and the redistribution of the fetal blood flow, contributing to stress adaptation [22]. Moreover, mechanical ventilation and septic shock are known to increase the production of vasopressin, as demonstrated in plasma and urine samples of newborns who received neonatal intensive cares [23,24]. Not to be forgotten that the oxidative stress secondary to pregnancy complications (i.e. pre-eclampsia, maternal insulin-dependent diabetes and growth-restriction) lead to lipid peroxidation, protein and DNA damage, both in the mother and the fetus. These mechanisms may have contributed to the ER overload and, finally, to the apoptosis or the premature cellular senescence [25,26].

*WFS1* expression has been fully characterized in the murine CNS. Wolframin presence has been confirmed in several CNS structures, comprising the amigdala, the hippocampal region, the hypothalamic magnocellular neurosecretory system, the brainstem nuclei, the globus pallidus, and the posterior caudate putamen [20,27]. Hilson and colleagues demonstrated that Wolfram patients exhibited abnormalities in various brain regions, including hypothalamus, pituitary, pons, inferior olivary nucleus, lateral geniculate nucleus, thalamus, cerebellum and optic nerve and optic tract [28].

The neuroimaging evolution of our patient at 1 year of life showed new pathological features such as reduced corpus callosus, moderate ventricular enlargement, asymmetric hippocampus and altered occipital peri-ventricular white matter signal. This suggests a neurodegenerative process affecting brain structures, known to be damaged by the loss of *WFS1*, but even more widely the white and gray matter. However, we can not exclude that the radiological findings as well as the mild psychomotor and mental retardation may be a consequence of prematurity and co-morbidities; In fact, disabilities affect roughly 30% of patients born between 29 and 32 weeks of gestational age and septic shock is an established cause of white matter damage [29,30].

The bilateral asymmetrical OA associated with the eyeball hypoplasia and the right orbital bone hypoplasia were evident by the first month of life, suggesting an alteration in the development rather than an involution of optic nerves.

In control mice, Wolframin is expressed in neurons and glial cells of the optic nerve, the SC, the SCN and the visual cortex, while in the retina it was expressed in all neuron subtypes [31]. In humans, it was found that Wolframin is abundant in retinal ganglion cells (RGCs), cell bodies and initial portion of axons, so RGCs could be affected by Wolframin disfunction both at level of the cell body or of the unmyelinated portion of the optic nerve. Such defect could lead to axonal transport deficits and/or perturbations of important cell functions, thus causing OA. Besides, the presence of wolframin in the optic nerve astrocytes suggests that *WFS1* mutations may affect their ability to provide nutrients to optic axons when they suffer from ER dysfunction [32].

In our patient, the presence of optic nerve hypoplasia with orbital bone hypoplasia long before term corrected age allowed new speculations about the underlying pathogenetic mechanisms, supported by recently published work. The vast majority of the critical processes involved in the retinal development occur between 24 weeks gestation and 3 to 4 months of postnatal life, while the axonal development of the optic tract occurs between 20–30 weeks of gestation [33]. A recent study by the Washington University Wolfram Study Group found a smaller intracranial volume, mainly due to reduced brainstem, cerebellum and optic radiation volume, starting from the earliest stages of the disease in Wolfram patients as compared to healthy controls and type 1 diabetic patients, [34], Such findings were considered more severe than neurobehavioural findings tested in their cohort of patients. The authors’ conclusions stated that these brain abnormalities exacerbated by a neurodegenerative process, originally result from a neurodevelopmental disruption dating back to the early stages of the CNS development. Similar conclusions were drawn thanks to post-mortem examination of Wolfram patients, as a discrepancy between the atrophic component of some brain regions (pons and medulla) and the neuronal loss was documented [28].

Heterozygous missense mutations in WFS1 have been linked to autosomal dominant low frequency neurosensory hearing impairment and psychiatric disorders [35]. Eight families with OA and deafness carrying a single, recurrent heterozygous missense mutation in WFS1 gene leading to reduced levels of mutant wolframin have been described [36]. More recently, Chaussenot et al. described a cohort of 96 Wolfram patients with a wide spectrum of phenotypes. In particular, about 14% of the patients showed late-onset symptoms (after 15 years of life) and one patient (WS9), affected by IUGR, cataract and psychomotor delay developed OA and hearing impairment by 6 months of life. Type 2 Wolfram syndrome, caused by mutations of the CISD2 gene on the chromosome 4q22, has been diagnosed in three large Jordanian families. The phenotype is similar to the type 1- syndrome although diabetes insipidus has not been described [37,38]. These reports underline our incomplete knowledge of wolframin functions.

## Conclusion

The analysis of our case reinforces the intriguing hypothesis that the *WFS1* plays a role in the development of neural structures, such as the optic nerve and the neuro-hypophyseal tract. After a careful examination of the patient and the extensive review of the literature, we speculate that this early and severe OA cannot be entirely explained by a progressive degenerative mechanism, although further studies are needed in order to identify all cerebral structures expressing Wolframin during embryogenesis and fully elucidate its role in the neurodevelopment.

## Consent

Written informed consent was obtained from both parents for the publication of this Case report. A copy of the written consent is available for review by the Editor-in-Chief of this journal.

No ethical approval was required for this case-report.
